# Artificial intelligence, emotional intelligence, and helplessness

**DOI:** 10.1016/j.bjorl.2025.101745

**Published:** 2025-12-05

**Authors:** Otavio B. Piltcher, Claire Hopkins

**Affiliations:** a Universidade Federal do Rio Grande do Sul, Department Otolaryngology Head and Neck Surgery, Porto Alegre, RS, Brazil; b Kings College London, Department Otolaryngology, London, United Kingdom

Some year ago, G Ullas, L McClelland, N S Jones, published a paper called Medically unexplained symptoms and somatization in ENT in this journal.^1^ The authors pointed that we were facing new approaches for medically unexplained symptoms, with a psychological base due to an association with anxiety and depression. Recently John W Ayres and colleagues^2^ published another article at JAMA comparing quality and empathy of physicians and artificial intelligence responses to patient’s questions. In practice despite of those interesting perspectives, we still see patients with this type of problem moving from office to office endlessly. This letter focuses on the physician’s central role to manage this problem.

The quest for answers to everything is an innate characteristic of human beings and clearly manifests between 3 and 5 years of age during the "why" phase. Whether definitive or not, true, or not these genuine inquiries, stemming from curiosity and a thirst for knowledge, invariably find some response. It cannot be asserted that this precocious experience guides future doubts and questions across various spheres. Nevertheless, the fact is that in doctor-patient relationships, everything repeats itself.

Evidently, this scenario is enriched by the inherent anxieties of fear of pain, mortality, and impatience on the part of patients. Alongside, on the part of the health professional there is the inability to cope with the helplessness that comes with a problem lacking an immediate solution. This equation turns into a negative vicious cycle that leads to numerous detriments for patients, who often find themselves undergoing various ineffective examinations and treatments with incurring costs and adverse effects. Furthermore, there’s the immeasurable consequence of fostering hope, which may initially seem positive, but ultimately acts as a boomerang bringing back frustration from expected failures. The consequence is an opposite effect, increasing symptoms and the inability to learn how to cope with certain limitations associated with the problem.

In otolaryngology, throat clearing and post-nasal drip are good examples of this situation. Those symptoms are very common but rarely have an etiology. Thus at least once, thorough physical examinations with complementary exams are mandatory. On the other hand, it is just astonishing the number of patients with this type of complaint that repeatedly perform the same investigations and even without a definite diagnosis lives with the sentence of having a label of chronic rhinitis and/or sinusitis and/or gastro esophageal reflux (GER). Consequently, they may be treated multiple times with antibiotics, prolonged topical and oral anti-allergy medicines and/or anti GER drugs. Unfortunately, with no improvement, just repeated periods of hope, expenses, and adverse effects, these patients search again for help, with the same questions and complaints. A true saga, a genuine pilgrimage.

It would be possible to discuss different thesis for the hypersensitivity in the pharyngeal wall of these patients, but that is not the goal of this text. The point is how to change this behavior that does not happen exclusively for this complain. Maybe the solution deserves new children’s educational strategies from the beginning building future individuals better prepare to accept the lack of solution for all problems. In the meantime, healthcare professionals must try to do their part. This is a significant challenge because, at times, as patients, they also share the same behavior, stemming from the same fears, anxieties and desire for immediate answers and solutions.

In an era of artificial intelligence (AI) this subject gets new insigths as there is an increasing expectation of having answers for everything. Interestingly, perhaps no different to humans, AI also seems to find it difficult admit the lack of an answer for any question. Notably, when asked to provide referenced information, ChatGPT frequently appears to provide false citations to support generated text. This appears to occur due to the limitations of the current language models, which use statistical methods to generate text that is similar to the large body of data that it is trained on. However, the training data itself may contain errors, and the model may not be able to distinguish this. It will create the most ‘probable’ answer, but cannot guarantee accuracy. Perhaps the inability to say I don't knowshows how far AI has evolved to mimic human responses.

After all physicians are neither magicians, nor bearers of divine powers with answers to all the situations that arise in everyday life. AI is already a reality, but the gap between theory and practice still huge. Engagement and adequate language are essential tools that compose emotional intelligence (EI).^3^ This ability if not naturally sprout should be taught, learned, and changed if needed. That seems to be the bridge to overcome this gap helping humans to cope adequately with their own frustrations and helplessness.

## ORCID ID

Otavio B. Piltcher: 0000-0003-3384-3664

Claire Hopkins: 0000-0003-3993-1569

## Declaration of competing interest

The authors declare no conflicts of interest.
